# Mucopolysaccharidosis type VI (Maroteaux-Lamy syndrome) in the pre-Columbian culture of Colombia

**Published:** 2014-06-30

**Authors:** Harry Pachajoa, Carlos Armando Rodriguez

**Affiliations:** 1 Centro de Investigaciones en Anomalías Congénitas y Enfermedades Raras. Universidad Icesi, Cali, Colombia; 2 Profesor Titular, Facultad de Salud, Universidad del Valle, Cali, Colombia

**Keywords:** Mucopolysaccharidosis VI, mucopolysaccharidoses, history of medicine, paleopathology, inborn genetic diseases

## Abstract

Mucopolysaccharidosis type VI or Maroteaux Lamy syndrome is an autosomal recessive lysosomal storage disorder resulting from a deficiency of arylsulfatase B, the clinical features include short stature, hepatosplenomegaly, dysostosis multiplex, stiff joints, corneal clouding, cardiac abnormalities, and facial dysmorphism, with intelligence usually normal. We present evidence of the possible existence of Maroteaux Lamy syndrome in pre-Columbian pottery 2000 years ago, in the Colombo-Ecuadorian Pacific coast of the Tumaco-Tolita culture.

## Abstract (es)

La mucopolisacaridosis tipo VI o síndrome de Maroteaux Lamy es una enfermedad de depósito lisosomal autosómica recesiva que resulta de una deficiencia de la enzima arilsulfatasa B, las características clínicas incluyen talla baja, hepatoesplenomegalia, disostosis múltiple, rigidez en las articulaciones, opacidad corneal, anomalías cardíacas, y dismorfismo facial, con inteligencia generalmente normal. Presentamos evidencia de la posible existencia de síndrome de Maroteaux Lamy en la cerámica precolombina desde hace 2000 años, en la costa pacífica colombo-ecuatoriana pertenecientes a la cultura Tumaco-Tolita.

## Introduction

Mucopolysaccharidosis type VI or Maroteaux Lamy syndrome is an autosomal recessive lysosomal storage disorder resulting from a deficiency of arylsulfatase B. Maroteaux *et al*. first described this disorder as a novel dysostosis associated with increased urinary excretion of chondroitin sulfate in 1963 1.

Clinical features and severity are variable, but usually include short stature, hepatosplenomegaly, dysostosis multiplex, stiff joints, corneal clouding, cardiac abnormalities, and facial dysmorphism, with intelligence usually normal 2.

**Figure 1:**
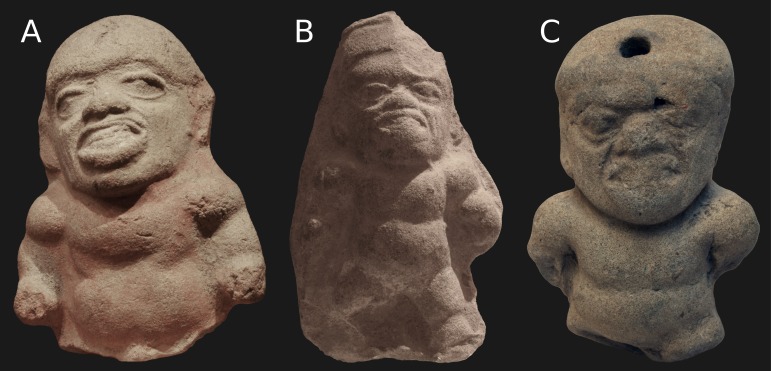
**A , B y C.** Pottery sample from the Tumaco-Tolita culture representing a possible case of Maroteaux Lamy syndrome.

We present a collection of pottery from the Tumaco-Tolita culture close to 2,000 years old with a possible representation of Maroteaux Lamy syndrome, which were evaluated by an archaeologist and a medical geneticist succeeded in identifying about 20 ceramic artifacts with representations of individuals with mucopolysaccharidosis type VI in about 10 museums and private collections in Ecuador and Colombia. The ceramic artifacts present phenotypical features like skeletal dysplasia, macrocephaly, mildly coarse facial features, broad mouth, prominent sternum, kyphosis, scoliosis (Fig. 1) probably constituting evidence of this disease in the pre-Hispanic Latin-American and past world. The differential diagnosis includes Hurler syndrome and various types of skeletal dysplasia.

The Tumaco-Tolita culture inhabited the region of the Pacific Colombo-Ecuadorian coast during the years 300 BCE to 600 CE. This culture was characterized for realistically representing the different pathologies affecting their population. In their pottery work, they show evidence of genetic illnesses like Down syndrome, achondroplasia, sirenomelia, and other congenital malformations 3 ^-^ 5. The representation of this syndrome in these populations could confirm the existence of this syndrome in these populations, and because of the high representation (20 ceramic pieces), it is suggested that it might have an increased prevalence, although it has not been possible to determine the population of these communities, and it is not possible to establish how many people had this disease, but the prevalence was almost certainly higher than currently reported for this syndrome (1 in 320,000 live newborns) 6. 

It is noteworthy that the figures represented are of young people or infants, clearly suggesting that the complications experienced by people with mucopolysaccharidosis type VI as cervical cord compression may have regulated the mortality of people with this disease, because at that time there was no orthopedic spine surgery. 

These types of pre-Hispanic indigenous communities, and especially the Tumaco-Tolita, lived in isolation, probably engaging in consanguineous marriages, as done by current indigenous Colombian communities, increasing the occurrence of recessive diseases and the possible existence of a founder effect.

Currently, the presence of this syndrome has been documented in indigenous communities in Cauca 7 and the presence of one or two founder effects it have been suggesteted in this area in the Colombian southwest 8. Because of the lack of genetic population studies in pre-Hispanic osseous remains of the Tumaco-Tolita, it is not possible to ensure that this population was related to the current residents of Cauca.
